# The prevalence of respiratory symptoms and diseases and declined lung function among foundry workers

**DOI:** 10.1186/s12995-024-00440-7

**Published:** 2024-10-24

**Authors:** Kirsi Koskela, Lauri Lehtimäki, Jukka Uitti, Panu Oksa, Antti Tikkakoski, Riitta Sauni

**Affiliations:** 1 Faculty of Medicine and Health Technology, Tampere University, P.O. Box 100, Tampere, FI-33014 Finland; 2 Finnish Institute of Occupational Health, Tampere, Finland; 3 Allergy Centre, Tampere University Hospital, Tampere, Finland; 4 Department of Clinical Physiology and Nuclear Medicine, Tampere University Hospital, Tampere, Finland

**Keywords:** Foundry work, Occupational exposure, Lung function, Occupational respiratory diseases

## Abstract

**Background:**

Foundry workers are occupationally exposed to a variety of inhalable chemical substances. Occupational exposure to vapors, gases, dusts, and fumes can lead to adverse health effects on the respiratory system and cause chronic respiratory diseases, such as interstitial lung diseases (ILDs), chronic obstructive lung disease (COPD), chronic bronchitis, and emphysema. Research on respiratory symptoms, diseases, and lung function in foundry workers over the past few decades has been limited. The aim of this cross-sectional study was to assess the prevalence of respiratory symptoms and diseases and declined lung function of current foundry workers, ex-foundry workers, and unexposed controls.

**Methods:**

We assessed respiratory symptoms, diseases, and lung function among 335 current foundry workers, 64 ex-foundry workers, and 161 unexposed controls. The cumulative dust exposure (mg-y) of each participant was calculated, and the median cumulative dust exposure according to the main places of exposure was determined.

**Results:**

A higher prevalence of chronic bronchitis, as reported in a questionnaire, was found among current and ex-foundry workers compared to unexposed controls, even after adjusting for pack-years of smoking (*p* = 0.009). Additionally, cough and wheezing in adulthood without respiratory infection, and chronic rhinitis symptoms were more common among current and ex-foundry workers compared to unexposed controls. These differences remained significant even after adjusting for pack-years of smoking and body mass index (BMI) (*p* = 0.007 and *p*  < 0.001, respectively). Impaired lung function was more prevalent among both ex-foundry workers (29.7%) and current foundry workers (15.5%) compared to the unexposed controls (8.7%), with the difference remaining significant even after adjusting for the pack-years of smoking and BMI (*p* = 0.009). According to the questionnaire, the number of physician-diagnosed cases of chronic obstructive pulmonary disease (COPD) or chronic bronchitis was unexpectedly low compared to the indications from the symptom questionnaire and lung function test results, suggesting a potential underdiagnosis. The prevalence of silicosis was low (0.8%) among current and ex-foundry workers.

**Conclusions:**

Respiratory symptoms are common among foundry workers. Current and ex-foundry workers exhibited lower lung function in spirometry compared to unexposed controls. There is a potential underdiagnosis of COPD and chronic bronchitis among foundry workers.

## Introduction

Occupational exposure to vapors, gases, dusts, and fumes can lead to various adverse health effects in the respiratory system and contribute to the burden of several chronic respiratory diseases [1, 2], including chronic obstructive pulmonary disease (COPD), chronic bronchitis, and emphysema [3, 4], as well as interstitial lung diseases (ILDs) [5, 6]. The population attributable fraction (PAF) due to occupational exposures is, for example, 14% for chronic obstructive pulmonary disease (COPD), 13% for chronic bronchitis, 26% for idiopathic pulmonary fibrosis, and essentially 100% for classical pneumoconiosis [1]. In addition to the nonmalignant diseases, occupational inhalable carcinogen exposures can cause cancer [7].

Foundry workers are exposed to a wide range of chemical substances associated with the materials and processes involved in foundry work. The processes in iron and steel casting consist of tasks such as molding, core-making, smelting, pouring, shake-out, and fettling. The occupational exposures include, for example, many kinds of dusts, including silica, metal fumes, carbon monoxide, binder compounds, and polycyclic aromatic hydrocarbons (PAHs) [8]. The composition and amount of inhalation exposure varies depending on the work tasks [9].

Due to their occupational exposure to silica, foundry workers are at risk of developing silicosis, a pneumoconiosis characterized by nodular changes [10]. In older studies from 1970s to 1980s, the prevalence of pneumoconiosis among foundry workers has ranged from 3.8 to 10.3% [11, 12]. Moreover, those with an extensive work history at the foundry have exhibited an even higher prevalence of silicosis [12]. A recent Swedish study published in 2022 reported that only 0.3% of foundry workers were diagnosed with silicosis, suggesting improvements in working conditions during the years [13]. Individuals exposed to silica are also at a heightened risk of mycobacterial infections such as tuberculosis [14]. Additionally, foundry workers have an elevated mortality rate from lung cancer [15, 16]. Occupational exposure to the inorganic compounds at the foundry can cause chronic bronchitis, airway obstruction, and COPD [11, 12, 17, 18]. Interestingly, a significantly increased COPD risk has been found in foundry workers even at cumulative silica exposures below 0.1 mg/m^3^ [19].

The mechanisms underlying the respiratory effects caused by occupational exposures are only partially understood. In our previous studies, we demonstrated that cumulative dust exposure in foundry work is associated with low-grade lung inflammation [20–22], which may precede the onset of a clinical disease. Our findings are supported by other studies where inflammatory reactions have been demonstrated in foundry workers [23–26].

The aim of the present study was to assess the prevalence of respiratory diseases, symptoms, and declined lung function among current and ex-foundry workers compared to unexposed controls in a cross-sectional setting.

## Methods

### Participants and study setup

A total of 617 workers participated in this study, which focused on two Finnish foundries owned by the same company: one was a steel casting foundry, and the other was an iron casting foundry. The study was initiated in 2004 and all employees of these foundries at that time point were invited to participate. Furthermore, to overcome the healthy worker bias, all former employees who were under the age of 70 and had a history of foundry work of at least 5 years, and had retired or changed workplace after January 1st, 1994, were invited as well. Assembly workers and white-collar workers from the same company without occupational exposure to foundry indoor air impurities formed the control group.

Participation was voluntary, and all participants gave their written informed consent. Ethical approval was obtained from the Ethics Committee of Tampere University Hospital (reference number R04168).

### Assessment of exposure

We assessed the cumulative exposure to inorganic dust and respirable silica during the working history as milligram-years (mg-y). In addition, potential cumulative exposure to organic dust was also evaluated as milligram-years (mg-y).

A job exposure matrix (JEM) was created based on the results of regular hygienic measurements at the workplace since the 1970s. In both foundries, measurements of total or inhalable inorganic dust had been carried out for decades with both stationary and personal samples. The combined results of stationary point and breathing zone measurements (dust and respirable silica) in different compartments from 1973 to 2004 are presented in our previous article [20].

The cumulative dust exposure of each participant was calculated by means of the JEM and the information about work assignments and work history during their career. All participants filled a questionnaire on working tasks and working history.

The median cumulative dust exposure according to the main places of exposure was also determined.

### Questionnaire on respiratory, eye, and skin symptoms and diseases

All participants completed a questionnaire regarding respiratory, skin, and eye symptoms and diseases diagnosed by physician, as well as their smoking habits. The questionnaire was based on the Finnish Tuohilampi series of questions [27], which was created for the purpose of population research.

In the questionnaire, participants indicated with a yes or no whether they had been diagnosed by a physician with chronic bronchitis, COPD, silicosis, pleural or parenchymal changes due to asbestos, asthma, allergic rhinitis, etc. An open question was also included for any other respiratory diseases not listed. The symptoms section included questions about cough, wheezing, mucus secretion, shortness of breath, and rhinitis symptoms, as well as whether these symptoms worsened at work. Additionally, participants were asked if they had ever experienced atopic eczema or allergic conjunctivitis.

According to the questionnaire, participants were considered to have chronic bronchitis based on the WHO definition if they answered “yes” to the following: (1) Question 4.2: Either “Have you had mucus secretion?” or “Have you had cough and mucus secretion?“, (2) Question 4.4: “Have you had mucus secretion almost daily for at least 3 months a year?” and (3) Question 4.5: “Have you had mucus secretion almost daily for at least 3 months for two consecutive years or longer?” Additionally, participants should not have a diagnosis of asthma or bronchiectasis.

### Spirometry

Spirometry was performed for all participants (Spiromaster, Medikro Oy, Finland and Vmax, Vyaire Medical, Illinois, USA). The two spirometers were compared to produce similar results. We measured forced vital capacity (FVC), forced expiratory volume in 1 s (FEV_1_), and their ratio (FEV_1_/FVC), and we used Finnish reference values [28].

### Statistical methods

SPSS 27 (IBM Corp. IBM SPSS Statistics for Windows, Version 27.0.1.0 Armonk, NY: IBM Corp.) software was used for statistical analysis. The normality of the distributions was evaluated by Q-Q-Plot. Kruskal-Wallis tests, Fischer-Freeman-Halton exact tests, Chi-square tests, ANOVA, or logistic regressions were used for between-group comparisons, and variable transformations were performed where appropriate.

In logistic regression models for respiratory, eye, and skin symptoms, adjustments were made for pack-years of smoking in relation to chronic bronchitis and atopic eczema, while the other symptoms were also adjusted for body mass index (BMI). Furthermore, the predicted and Z-score values of lung function variables as well as the proportion of impaired lung function were adjusted for pack-years of smoking and BMI, and in the case of absolute lung function variables, adjustments were made for age as well. A p-value of < 0.05 was considered statistically significant.

## Results

### Study population and subjects’ characteristics

**Fig. 1:**
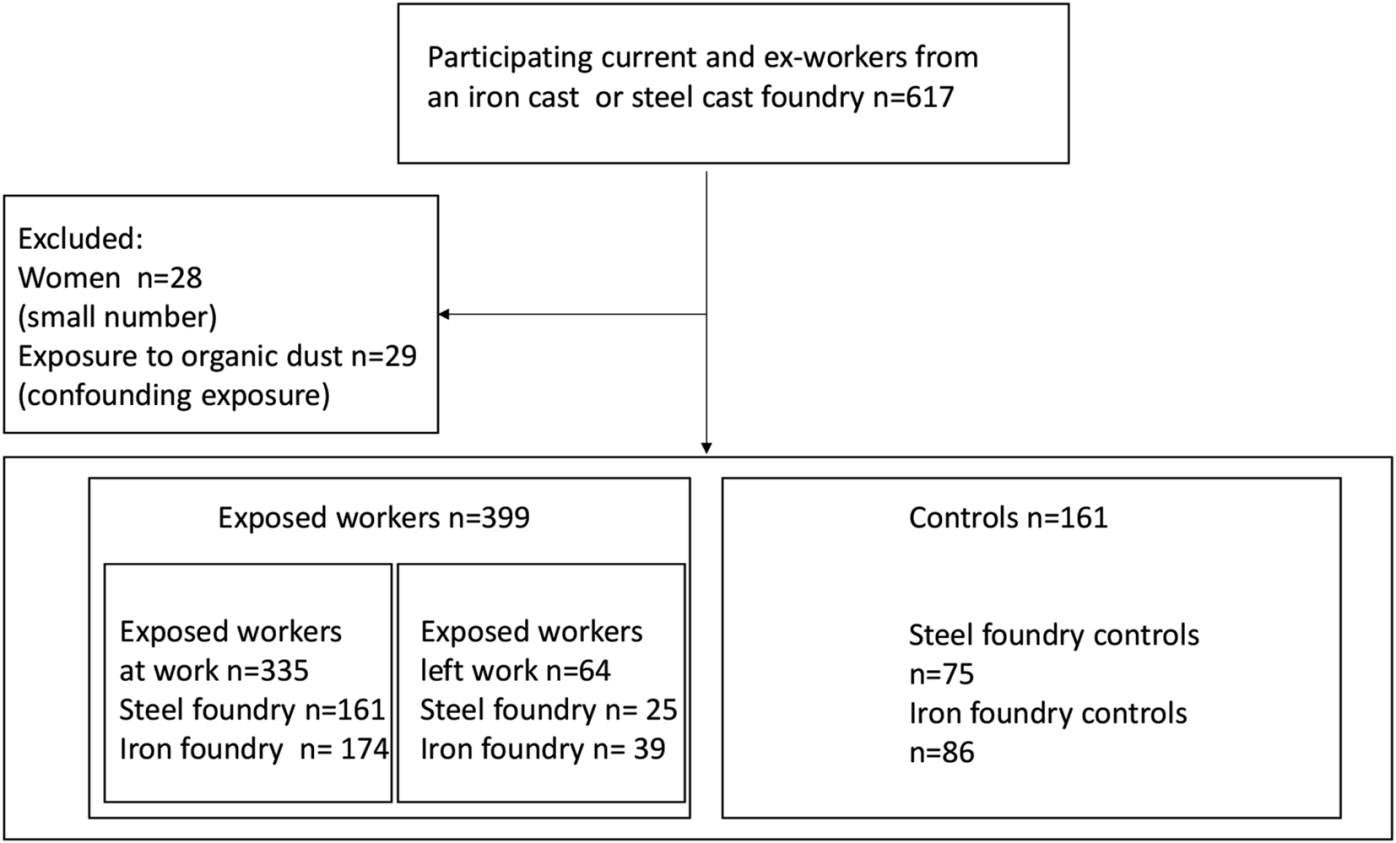
The flowchart of the study

Figure 1 shows the flowchart of our study. Altogether, 617 exposed or unexposed workers participated the study. In the further analyses, the exclusion criteria were as follows: female gender (*n* = 28), since the subpopulation was quite small and the percentage of female subjects varied significantly in different subgroups (1.8–9.5%), and subjects with possible confounding occupational exposures in the foundry (like organic dust) (*n* = 29).

The final study population included 560 participants: 399 exposed, comprising 335 subjects who were currently working in the foundry (current foundry workers) and 64 subjects who had left foundry work (ex-foundry workers), and 161 unexposed controls (Fig. 1).

Table 1 shows the characteristics of the study population. The age, body mass index (BMI), smoking status, and pack-years of smoking varied significantly between the three study groups. Notably, ex-foundry workers exhibited a higher age, BMI, and pack-years of smoking but had a lower proportion of current smokers compared to the other study groups.

**Table 1:** Characteristics of the study population

	Unexposed controls (*n* = 161)	Current foundry workers (*n* = 335)	Ex-foundry workers (*n* = 64)	*p*-value
Age, years mean (SD)	44.2 (10.2)	43.9 (11.5)	61.6 (6.6)	< 0.001*^a^
Smoking status number (percentage)				
current smoker	57 (35%)	132 (39%)	11 (17%)	< 0.001*^b^
ex-smoker	36 (22%)	93 (28%)	29 (45%)	
never-smoker	68 (42%)	110 (33%)	24 (38%)	
Pack-years of smoking among current smokers and ex-smokers mean (SD)	8.4 (11.8)	10.8 (13.0)	15.1 (19.9)	0.048*^a^
BMI (kg/m^2^) mean (SD)	26.5 (3.1)	27.3 (3.6)	28.1 (4.5)	0.016*^a^

The results are presented as the mean value (SD) or number (%)

^a^Kruskal-Wallis test, ^b^Chi-square test

### Cumulative dust exposure

Current foundry workers had an average exposure of 18.0 years (median of 17.0 years) in the foundry, while ex-foundry workers had an average exposure of 28.1 years (median of 31.0 years). The median cumulative dust exposure for current foundry workers was 53.00 mg-y (interquartile range: 25.00–146.00 mg-y), while for ex-foundry workers, the median cumulative exposure was higher at 123.00 mg-y (interquartile range: 69.25–234.00 mg-y).

Table 2 shows the median cumulative dust exposure and interquartile range categorized by the main place of exposure for both current foundry workers and ex-foundry workers. The molding shop workers among the current foundry workers had the highest median cumulative dust exposure, with a median of 77.00 mg-y (interquartile range: 35.00–199.75 mg-y). Similarly, among ex-foundry workers, those who had worked in the molding shop had the highest median cumulative dust exposure, with a median of 194.00 mg-y (interquartile range: 141.00–245.50 mg-y).

Significant variations in median cumulative dust exposure were observed based on the main place of exposure for both current foundry workers (*p* < 0.001) and ex-foundry workers (*p* < 0.001).

**Table 2:** The median cumulative dust exposure according to the main place of exposure in current foundry workers and ex-foundry workers

	Cumulative dust exposure (mg-y)				*p*-value
	Smeltery at work *n* = 40 left work *n* = 6	Molding shop at work *n* = 152 left work *n* = 21	Fettling shop at work *n* = 98 left work *n* = 17	Other at work *n* = 44 left work *n* = 20	
Current foundry workers #	75.00 (27.00–146.75)	77.00 (35.00–199.75)	37.00 (19.50–101.00)	36.00 (12.50–70.00)	< 0.001*^a^
Ex-foundry workers	121.00 (95.25–170.50)	194.00 (141.00–245.50)	121.00 (69.50–428.50)	66.00 (41.00–86.50)	< 0.001*^a^

The results are presented as the median value and interquartile range

# The main place of exposure was missing for one current foundry worker

^a^Kruskal-Wallis test

### Lower respiratory tract diseases diagnosed by a physician, as reported in the questionnaire

Table 3 illustrates the prevalence of lower respiratory tract diseases diagnosed by a physician, as reported in the questionnaire in the unexposed controls, current foundry workers, and ex-foundry workers. The prevalence of chronic obstructive lung disease (COPD) showed variation among the study groups (*p* = 0.004), since all cases were found exclusively in current or ex-foundry workers.

Additionally, the prevalence of pleural or lung parenchymal changes attributed to asbestos, silicosis, and mixed dust pneumoconiosis differed significantly between the study groups (*p* = 0.043, *p* = 0.036, and *p* = 0.013, respectively).

In the study population, the prevalence of silicosis, mixed dust pneumoconiosis, and pleural or lung parenchymal changes caused by asbestos was low among both current and ex-foundry workers. Only one case of pleural plaques or lung parenchymal changes caused by asbestos was registered in the unexposed controls.

No significant differences were observed in the prevalence of chronic bronchitis, asthma, pleuritis, pneumonia, sarcoidosis, or tuberculosis among the study groups. One case of tuberculosis was found, but it was not related to dust exposure as it was diagnosed in an unexposed control.

**Table 3:** Lower respiratory diseases diagnosed by a physician at baseline in the unexposed controls, current foundry workers, and ex-foundry workers

	Unexposed controls (*n* = 161)	Current foundry workers (*n* = 335)	Ex-foundry workers (*n* = 64)	*p*-value
Chronic bronchitis	3 (1.9%)	7 (2.1%)	2 (3.1%)	0.824 ^b^
Chronic obstructive pulmonary disease (COPD)	0 (0%)	4 (1.2%)	4 (6.3%)	0.004*^b^
Asthma	7 (4.3%)	14 (4.2%)	5 (7.8%)	0.402^b^
Pleural or lung parenchymal changes caused by asbestos	1 (0.6%)	10 (3.0%)	4 (6.3%)	0.043*^b^
Pleuritis	1 (0.6%)	5 (1.5%)	2 (3.1%)	0.304^b^
Silicosis	0 (0%)	1 (0.3%)	2 (3.1%)	0.036*^b^
Pneumonia	20 (12.4%)	36 (10.7%)	11 (17.2%)	0.342^a^
Sarcoidosis	1 (1%)	3 (0.9%)	0 (0%)	1.000^b^
Mixed dust pneumoconiosis	0 (0%)	0 (0%)	2 (3.1%)	0.013*^b^
Tuberculosis	1 (0.6%)	0 (0%)	0 (0%)	0.403^b^

^a^Chi-square test, ^b^Fischer-Freeman-Halton exact test

### Questionnaire on respiratory, eye, and skin symptoms

Table 4 presents the prevalence of respiratory, eye, and skin symptoms, as indicated by the questionnaire, among the unexposed controls, current foundry workers, and ex-foundry workers.

The study groups demonstrated a significant variation in the prevalence of chronic bronchitis (*p* < 0.001), even after adjusting for pack-years of smoking (*p* = 0.009). Remarkably, ex-foundry workers had the highest prevalence of chronic bronchitis (28.1%), followed by current foundry workers (12.2%).

Ex-foundry workers demonstrated the highest prevalence of cough and wheezing in adulthood without respiratory infection (14.1%), surpassing both current foundry workers (7.5%) and the unexposed controls (1.9%). These differences between the study groups were significant (*p* = 0.001), even after adjusting for pack-years of smoking and BMI (*p* = 0.007). However, there were no significant differences in the prevalence of attacks of shortness of breath and wheezing among the study groups (*p* = 0.375).

The prevalence of chronic rhinitis symptoms was distinctly high across all study groups, ranging from 26.1 to 50.0%. However, there were significant differences observed between the study groups (*p* = 0.002), even after adjusting for pack-years of smoking (*p* < 0.001). Both ex-foundry workers and current foundry workers reported a higher prevalence of chronic rhinitis symptoms compared to the unexposed controls. In the unexposed controls, the chronic rhinitis symptoms were associated with allergic rhinitis symptoms triggered by factors such as pollen or animals. However, current foundry workers and ex-foundry workers experienced chronic rhinitis symptoms that were unrelated to allergens. There were no significant differences found in the prevalence of allergic conjunctivitis symptoms or atopic eczema between the study groups.

We evaluated the associations between age and the different symptoms presented in Table 4 in the unexposed controls. Since there were no significant associations between age and different symptoms, age was not included in the further analyses.

**Table 4:** The prevalence of respiratory, eye, and skin symptoms based on the questionnaire in unexposed controls, current foundry workers, and ex-foundry workers

	Unexposed controls (*n* = 161)	Current foundry workers (*n* = 335)	Ex-foundry workers (*n* = 64)	*p*-value	*p*-value adjusted
Chronic bronchitis, n %	15 (9.3%)	41 (12.2%)	18 (28.1%)	< 0.001*^a^	0.009* ^c^
Attacks of shortness of breath and wheezing, n %	12 (7.5%)	28 (8.4%)	8 (12.5%)	0.375^a^	0.559^d^
Cough and wheezing in adulthood without respiratory infection, n %	3 (1.9%)	25 (7.5%)	9 (14.1%)	0.001*^b^	0.007*^d^
Chronic rhinitis symptoms n %	42 (26.1%)	124 (37.0%)	32 (50.0%)	0.002*^a^	< 0.001*^d^
Allergic rhinitis symptoms (pollen, animals etc.) n %	42 (26.1%)	78 (23.3%)	17 (26.6%)	0.706^a^	0.894^d^
Allergic rhinitis diagnosed by a physician n	23 (14.3%)	19 (5.7%)	4 (6.3%)	< 0.001* ^b^	^−^
Rhinitis symptoms work-related n	19/42 (45.2%)^e^	61/124 (49.2%)^e^	-	0.115^a^	0.178^d^
Allergic conjunctivitis symptoms n %	30 (18.6%)	62 (18.5%)	14 (21.9%)	0.823^a^	0.570^d^
Allergic conjunctivitis diagnosed by a physician n	15 (9.3%)	18 (5.4%)	4 (6.3%)	0.136^b^	-
Atopic eczema n %	38 (23.6%)	74 (22.1%)	19 (29.7%)	0.587^a^	0.802^c^

^a^Chi-square test, ^b^Fischer-Freeman-Halton Exact test, ^c^ logistic regression adjusted for pack-years of smoking, ^d^ logistic regression adjusted for pack-years of smoking and BMI, ^e^ number of cases with work-related rhinitis symptoms compared to the cases of chronic rhinitis symptoms

In a subpopulation of subjects without asthma, chronic obstructive lung disease, or chronic bronchitis diagnosed by a physician, the prevalence of chronic bronchitis varied significantly between the study groups (*p* = 0.002), even after adjusting for pack-years of smoking (*p* = 0.017). The prevalence of chronic bronchitis was 28.6% (16 cases) in ex-foundry workers, 12.5% (39 cases) in current foundry workers, and 9.9% (15 cases) in the unexposed controls. Additionally, the prevalence of cough and wheezing in adulthood, without respiratory infection, also exhibited significant variation among the study groups (*p* = 0.037), but this difference did not remain significant (*p* = 0.065) after adjusting for pack-years of smoking and body mass index (BMI). The occurrence of attacks of shortness of breath and wheezing did not show significant variation between the study groups (*p* = 0.723).

### Lung function

Table 5 shows the lung function variables and proportion of subjects with impaired lung function in the unexposed controls, current foundry workers, and ex-foundry workers. All lung function variables varied statistically significantly between the different study groups even after adjusting for pack-years of smoking and BMI, and for absolute lung function variables also for age.

**Table 5:** Lung function variables and proportions of subjects with impaired lung function in the unexposed controls, current foundry workers, and ex-foundry workers

	Unexposed controls *n* = 160	Current foundry workers *n* = 335	Ex-foundry workers *n* = 64	*p*-value	*p*-value adjusted
FVC (l)	5.30 (0.80)	5.11 (0.84)	4.26 (0.68)	< 0.001*^c^	0.002*^d^
FVC % predicted	98.13 (11.17)	94.31 (11.62)	88.91 (12.57)	< 0.001*^c^	< 0.001*^e^
FVC Z-score	-0.15 (0.96)	-0.46 (0.98)	-0.76 (0.88)	< 0.001*^c^	0.004*^e^
FEV_1_(l)	4.33 (0.72)	4.08 (0.69)	3.32 (0.68)	< 0.001*^c^	< 0.001*^d^
FEV_1_% predicted	102.11 (13.31)	96.04 (12.60)	91.81 (17.49)	< 0.001*^b^	< 0.001*^e^
FEV_1_ Z score	0.18 (1.08)	-0.32 (1.01)	-0.53 (1.12)	< 0.001*^c^	< 0.001*^e^
FEV_1_/FVC	0.82 (0.06)	0.80 (0.06)	0.78 (0.09)	< 0.001*^b^	0.004*^d^
FEV_1_/FVC % predicted	103.84 (7.47)	101.71 (7.64)	102.59 (11.25)	0.002*^b^	0.009*^e^
FEV_1_/FVC Z score	0.55 (1.05)	0.24 (1.07)	0.37 (1.56)	0.002*^b^	0.006*^e^
Impaired lung function FVC Z score, FEV_1_ Z score or FEV_1_/FVC Z score < -1.65	14 (8.7%)	52 (15.5%)	19 (29.7%)	< 0.001*^a^	0.009*^f^

^a^ Chi-square test, ^b^ Kruskal-Wallis test, ^c^ ANOVA, ^d^ ANOVA adjusted for BMI, pack-years of smoking, and age, ^e^ ANOVA adjusted for BMI and pack-years of smoking, ^f^ logistic regression adjusted for BMI and pack-years of smoking

There were significant differences in the prevalence of impaired lung function among the study groups (*p* < 0.001) even after adjusting for the number of pack-years of smoking and BMI (*p* < 0.009). The prevalence of impaired lung function was higher among ex-foundry workers (29.7%) and current foundry workers (15.5%) compared to the unexposed control group (8.7%).

In a subpopulation of individuals without asthma, impaired lung function was observed in 16 cases (27.1%) among ex-foundry workers, 48 cases (15.0%) among current foundry workers, and only 13 cases (8.4%) among the unexposed controls. The difference between the study groups remained statistically significant (*p* = 0.002) even after adjusting for the number of pack-years of smoking and BMI (*p* = 0.019).

## Discussion

The main findings of this study were a significant association between occupational dust exposure and a higher prevalence of chronic bronchitis, cough and wheezing, and chronic rhinitis symptoms. Impaired lung function was more prevalent among both ex-foundry workers and current foundry workers in comparison to the unexposed controls, even after adjusting for the number of pack-years of smoking and BMI. According to the questionnaire the number of COPD or chronic bronchitis diagnosed by a physician was unexpectedly low compared to the results from the symptom questionnaire or lung function tests. Additionally, the prevalence of silicosis was only 0.8% among current and ex-foundry workers.

Foundry workers are exposed to a range of occupational inhalable hazards, including dusts, silica, metal fumes, gases, and elevated levels of ultrafine particles (PM) [8, 29]. Silicosis is a well-recognized respiratory disease among foundry workers that results from exposure to respirable silica. In a large Finnish foundry study in the 1950s, the prevalence of silicosis and lung fibrosis based on radiographs was 6.5%, and most cases (68%) were registered in the group who had been exposed for over 20 years [30]. About 20 years later, a comprehensive study of Finnish foundry workers in the 1970s found pneumoconiosis in 3.8% of those exposed based on radiographs [11]. In the current study, the prevalence of silicosis (0.8%), mixed dust pneumoconiosis (0.5%), and pleural or lung parenchymal changes caused by asbestos (3.5%) among exposed workers, as determined by a questionnaire, was found to be low.

In addition to the above-mentioned Finnish studies, a study conducted in an iron and steel foundry in Canada in the 1980s found pneumoconiosis in 4.8% of foundry workers [31] In a South African foundry, the overall prevalence of pneumoconiosis was 10.3%, increasing to 38.0% among workers with more than 15 years of work experience [12]. A study in American foundries revealed that 9.6% of foundry workers had silicosis [32]. In the 1990s, 7.5–8.8% of foundry workers in Taiwan had pneumoconiosis [33, 34], and in an American automotive foundry, signs of silicosis were found in 2.9% of foundry workers [10]. Participants with longer working histories at the foundry exhibited a higher prevalence of silicosis, with rates reaching up to 12.0% for those with over 30 years of experience [10]. In a Brazilian study published in 2007, pneumoconiosis was found in 4.5% of foundry workers [35]. In the aforementioned studies, chest radiographs were used to diagnose pneumoconiosis or silicosis, which could potentially lead to misinterpretation or underdiagnosis, as the specific type of pneumoconiosis can be challenging to identify based solely on chest x-rays. In addition to silica dust exposure, workers at the foundry may have typically been exposed to asbestos, which could have led to possible pneumoconiotic changes [36].

In a recently published study conducted in Sweden, silicosis was found in 6 out of 1,752 foundry workers (0.3%) which is quite similar in magnitude to the prevalence found in our study (0.8%) [13]. The findings suggest that the risk of silicosis among foundry workers in developed countries has significantly decreased over the past decades, and this is attributed to the lowering of the occupational safety limit for silica and substantial enhancements in working conditions. However, it is important to note that the risk of silicosis has not been eliminated.

In the present study, chronic bronchitis was found in 12.2% of current foundry workers and up to 28.1% ex-foundry workers based on the symptom questionnaire. The difference between study groups was significant even after adjusting for pack-years of smoking. In the previous foundry study in Finland, chronic bronchitis was identified in 16% of foundry workers based on a questionnaire that focused on respiratory symptoms. Additionally, it was observed that chronic bronchitis occurred more frequently among participants with dusty occupations [11]. Previously, in a small sample of iron and steel foundry workers, chronic bronchitis was found in 17.7% of the participants, but cough or phlegm was found more often (29.0% and 38.7%, respectively) [31].

In a study conducted in a rapidly developing country, exposed foundry workers experienced higher rates of cough (38.3%), phlegm production (30.9%), and dyspnea (20.9%) compared to the controls [18]. Foundry workers with high current dust exposure have reported a higher prevalence of cough (25.6%) compared to workers with moderate or light dust exposure (12.6%), despite there being no significant difference in the prevalence of chronic bronchitis (7.0% and 4.2%, respectively) [32]. Symptoms of chronic bronchitis have also been found to be associated with the duration of exposure at the foundry [35]. In a study conducted in Turkey, cough was reported in 16.8% of foundry workers and phlegm in 23.1%, but breathlessness was a less common symptom (9.1%) [37]. Based on our study, the prevalence of chronic bronchitis remains overrepresented, particularly among ex-foundry workers.

In the current study, ex-foundry workers in particular exhibited a high prevalence of cough and wheezing in adulthood without respiratory infection. Additionally, current foundry workers showed an almost fourfold prevalence of these symptoms compared to the unexposed controls. The differences remained significant even after adjusting for pack-years of smoking and BMI. We could not demonstrate significant differences in the prevalence of attacks of shortness of breath and wheezing, which are more indicative for asthma, among the study groups. Foundry workers appear to frequently report lower respiratory tract symptoms, primarily indicating chronic bronchitis and chronic obstructive pulmonary disease.

Both the current and ex-foundry workers reported a higher prevalence of chronic rhinitis symptoms compared to the unexposed controls. The prevalence of chronic rhinitis symptoms was highest among ex-foundry workers, with half of them reporting these symptoms. In the unexposed controls, the chronic rhinitis symptoms were associated with allergic rhinitis symptoms triggered by animals or pollen, etc. More than half of both current and former foundry workers with chronic rhinitis symptoms experienced symptoms of allergic rhinitis. However, they also suffered from chronic rhinitis symptoms that were not related to allergens. Approximately half of the current foundry workers with chronic rhinitis symptoms indicated that their rhinitis symptoms were work-related. Previously, in a Swedish study, nasal symptoms and signs were more prevalent among exposed foundry workers compared to the controls since exposed foundry workers experienced nasal symptoms very often during the week immediately preceding the nasal examination (74%) [38]. In addition to affecting the lower respiratory tract, foundry work also triggers irritation in the nasal cavity leading to chronic rhinitis symptoms.

We found that all lung function variables varied significantly between the different study groups even after adjusting for pack-years of smoking and BMI, and for absolute lung function variables also for age. Exposed workers, either current or ex-foundry workers, exhibited lower lung function compared to the unexposed controls. Moreover, both current and ex-foundry workers exhibited a higher prevalence of impaired lung function compared to the controls, with ex-foundry workers showing a 3.4-times higher prevalence than unexposed controls. Previously, exposed foundry workers have shown a lower FEV_1_/FVC compared to unexposed controls even after adjusting for pack-years of smoking and age [18]. In another study, exposed foundry workers were found to have a lower FEV_1_ compared to controls, even after adjusting for age, height, and smoking habits [31]. Furthermore, the duration of foundry work has been linked to a more pronounced decline in FVC and FEV_1_, even after adjusting for age, height, and smoking [34]. When measuring lung function before and after a work shift, silica exposure has been associated with a decrease in both FVC and FEV_1_ in foundry workers [39]. In the 1970s in Finland, no association between dust exposure and impairment of lung function could be found in foundry workers. It was estimated that the selective removal of workers from dusty jobs may have influenced the findings. In addition, foundry workers were divided into three dust exposure categories based on the current dust exposure rather than cumulative dust exposure, which probably influenced the results as well [11].

The prevalence of chronic obstructive lung disease or chronic bronchitis diagnosed by a physician was surprisingly low compared to the findings from the questionnaire and lung function tests. In a subpopulation of subjects without asthma, chronic obstructive lung disease, or chronic bronchitis diagnosed by a physician, the prevalence of chronic bronchitis based on the symptom questionnaire was found in 70 participants, with 55 of them being exposed subjects. Moreover, in a subgroup of individuals without asthma, impaired lung function was observed in 77 participants, with 55 of them being exposed subjects. Our findings suggest a potential underdiagnosis of COPD and chronic bronchitis especially among exposed foundry workers. It is possible that mild respiratory symptoms that do not significantly impact work ability may go unnoticed during the health surveillance of foundry workers.

In a recently published study, foundry workers were reported to have a significantly increased morbidity risk for COPD, bronchitis, and pneumonia. Each participant’s cumulative silica exposure was assessed using exposure measurement data and job titles. The exposure data were then integrated with morbidity data obtained from the patient register. Interestingly, a significantly increased COPD risk was found at cumulative silica exposures below 0.1 mg/m^3^ [19].

The strength of our study was the large study population including both current and ex-foundry workers who were compared with the unexposed controls. We were able to adjust for BMI, age, and pack-years of smoking when appropriate, and we conducted subgroup analyses. The cumulative dust exposure for each participant was calculated by industrial hygienists using the job exposure matrix (JEM) and the information about work history and different tasks of the participant’s career. We were able to report mean cumulative dust exposure levels at four different main places of exposure, namely the smeltery, molding shop, fettling shop, and other in both current and ex-foundry workers, and thus our results can be generalized to other foundries with similar occupational dust exposure.

A weakness of our study is the lack of reliable information regarding the participation rate. The study was carried out in collaboration with the occupational health services of the participating foundries, which were responsible for recruiting both exposed current and ex-foundry workers and the unexposed subjects for this study. The research team did not have access to the annual count of workers and their identities from 1994 to 2004, which would have allowed for the calculation of the participation rate. As a result, the participation rate was not documented. There is a possibility that some selection bias may have occurred, as individuals in a better health condition who are interested in promoting their health in general may have been more eager to participate in this type of study. It is possible that especially ex-foundry workers with more severe health conditions did not participate. Ex-foundry workers who were under the age of 70 at the time of the study were invited. It is important to note that some adverse health effects caused by occupational exposure such as silicosis can be diagnosed even later in life. Nevertheless, we were able to assemble a sizable study population encompassing a broad spectrum of cumulative dust exposure, as well as diverse age distributions.

The healthy worker selection bias may have had an influence on our results by underestimating the symptoms, diseases, and the decrease in lung function in exposed subjects and diminishing the differences between exposed subjects and the unexposed controls. Before starting to work at the foundry, foundry workers undergo a pre-employment health examination. It is improbable that individuals with a previously diagnosed chronic respiratory disease would engage in physically demanding foundry work. Foundry workers also undergo fixed-period health examinations, during which attention is also given to the respiratory symptoms and diseases. Workers with chronic respiratory diseases that impact their ability to work are often reassigned to tasks with reduced occupational exposure within the company or seek other job opportunities. However, we were unable to gather information on how many exposed participants had changed their work assignments due to health issues prior to the study.

We were not able to perform a bronchodilatation test for all those participants that had a decreased lung function at spirometry, and thus we were not able to specify possible asthma or COPD for those having impaired lung function. Based on the symptom questionnaire, the prevalence of attacks of shortness of breath and wheezing was twofold compared to the asthma cases diagnosed by a physician, indicating that there is a potential underdiagnosis of asthma. Nevertheless, it is improbable that individuals experiencing asthma attacks would go unnoticed during routine health examinations or comprehensive occupational health services.

We did not include chest radiographs as part of our study methods; instead, we inquired about diagnoses through a questionnaire. As part of the routine health examinations at the occupational health services, workers regularly undergo chest radiography. Therefore, it is unlikely that any potential pneumoconiosis that could be evident in a chest radiograph would go unnoticed. Asbestos-related diseases were combined in our questionnaire, and thus we were not able to differentiate between pleural plaques and asbestosis. Regardless, the prevalence of asbestos-related diseases was low (2.7%), and these cases were most likely pleural plaques.

The current study contributes important insights into respiratory symptoms, diseases, and lung function among foundry workers in an industrialized environment where significant efforts have been made to enhance occupational hygiene and working conditions. In addition, our foundry study builds on previous research in Finland [11, 30], providing a valuable historical perspective stretching over 50 years.

Over the years, the working conditions at foundries have improved, with revisions to the occupational safety limits for silica leading to increased demand to reduce occupational dust exposure. In terms of health effects, the focus of foundry work has been particularly on exposure to silica. In addition to respiratory diseases, an increased risk for cardiovascular disease among foundry workers has also been reported [40]. Exposure to silica also carries a risk for autoimmune disorders [41] as well as lung cancer [15, 16]. However, attention is also required for exposure to other foundry air impurities, and further studies are needed to evaluate which working conditions at the foundries are safe in order to prevent adverse health effects.

The mechanisms underlying the health effects are only partially understood. Our previous studies have shown that foundry work triggers subclinical inflammation, which precedes the onset of a clinical disease [20–22]. Our findings are consistent with other studies where markers of inflammation and their association with occupational exposure among foundry workers have been evaluated [23–26].

In conclusion, foundry workers are exposed to a wide range of occupational inhalable hazards that can lead to adverse health effects. Despite improvements in working conditions, respiratory symptoms are common among foundry workers, and occupational dust exposure contributes to a decline in lung function. There is a potential underdiagnosis of COPD and chronic bronchitis among foundry workers. Further attention is required for the occupational exposure at the foundries, and there is still a need to minimize the inhalation exposure of foundry workers. Additional studies are necessary to investigate the health of foundry workers also in contemporary environments, monitor the occupational exposure levels at foundries, and explore the mechanisms underlying the health effects.

## Data Availability

No datasets were generated or analysed during the current study.

## Abbreviations

BMI: Body mass index
COPD: Chronic obstructive pulmonary disease
FVC: Forced vital capacity
FEV_1_: Forced expiratory volume in 1 s
FEV_1_/FVC: Forced expiratory volume in 1 s/ forced vital capacity
mg-y: Milligram years
SD: Standard deviation
